# Water Extract from Brown Strain of *Flammulina velutipes* Alleviates Cisplatin-Induced Acute Kidney Injury by Attenuating Oxidative Stress, Inflammation, and Autophagy via PI3K/AKT Pathway Regulation

**DOI:** 10.3390/ijms24119448

**Published:** 2023-05-29

**Authors:** Ya-Ni Chou, Min-Min Lee, Jeng-Shyan Deng, Wen-Ping Jiang, Jaung-Geng Lin, Guan-Jhong Huang

**Affiliations:** 1 Department of Chinese Pharmaceutical Sciences and Chinese Medicine Resources, College of Chinese Medicine, China Medical University, Taichung 404, Taiwan; yanichoucmu@gmail.com; 2 Department of Food Nutrition and Healthy Biotechnology, Asia University, Taichung 413, Taiwan; leemm@asia.edu.tw (M.-M.L.); dengjs@asia.edu.tw (J.-S.D.); 3 Department of Pharmacy, Chia Nan University of Pharmacy and Science, Tainan 717, Taiwan; wpjiang@gm.cnu.edu.tw; 4 School of Chinese Medicine, College of Chinese Medicine, China Medical University, Taichung 404, Taiwan; jglin@mail.cmu.edu.tw; 5 Chinese Medicine Research Center, China Medical University, Taichung 404, Taiwan

**Keywords:** *Flammulina velutipes*, cisplatin, acute kidney injury, autophagy, PI3K/AKT pathway

## Abstract

One of the most popular edible mushrooms in the world, *Flammulina velutipes*, has been shown to possess pharmacological properties such as anti-inflammatory and antioxidant properties. However, the potential activity of the brown strain of *F. velutipes*, a hybrid between the white and yellow strains, has not been thoroughly investigated. Numerous studies have been conducted in recent years to determine whether natural products can aid in improving or treating kidney diseases. In this study, we focused on the renoprotective effects of the brown strain of *F. velutipes* on cisplatin-induced acute kidney injury (AKI) in mice. Mice were pretreated with water extract from the brown strain of *F. velutipes* (WFV) from day 1 to day 10, with a single-dose intraperitoneal injection of cisplatin on day 7 to induce AKI. Our results demonstrated that WFV administration resulted in a reduction in weight loss and the amelioration of renal function and renal histological changes in mice with cisplatin-induced AKI. WFV improved antioxidative stress and anti-inflammatory capacity by increasing antioxidant enzymes and decreasing inflammatory factors. The expression of related proteins was determined via Western blot analysis, which showed that WFV could improve the expression of apoptosis and autophagy. We used the PI3K inhibitor Wortmannin and found that WFV achieved a protective effect by modulating the PI3K/AKT pathway and the expression of autophagy. Overall, WFV as a natural substance could be used as a new therapeutic agent for AKI.

## 1. Introduction

Acute kidney injury (AKI) is an increasingly prevalent disease associated with morbidity and mortality, affecting 13.3 million people worldwide, killing 1.7 million people worldwide each year, and lacking effective treatment beyond supportive care [1]. The diagnosis of AKI is clinically determined by serum creatinine levels and urine output [2]. Intrarenal factors are a major cause of AKI and may be due to nephrotoxic drugs [3] such as cisplatin, a commonly used chemotherapeutic agent, the side effects of which can cause nephrotoxicity [4].

Cisplatin induces apoptosis by cross-linking with the purine bases of cancer cells [5]. However, cisplatin is excreted through the kidneys, and platinum accumulates in the kidneys, which can cause proximal tubular damage, oxidative stress, and renal vascular damage, leading to acute kidney injury [6]. Cisplatin-induced AKI is typically accompanied by an increase in reactive oxygen species (ROS) production, which results in the accumulation of lipid peroxides and the inhibition of antioxidant systems [7,8].

It is believed that the main mechanism by which renal tubular epithelial cells (RTECs) are damaged by cisplatin is apoptosis, and it was found that RTECs rely specifically on autophagy to maintain constancy and respond to stress [9]. Many natural substances have been reported to potentially improve cisplatin-induced AKI, such as pomegranate rind hydroalcoholic extract [10] and *Cordyceps cicadae* mycelia water extract [11]. However, natural extracts have been studied more often for their antioxidant and anti-inflammatory properties, and it is still not well understood whether they affect autophagy. There is mounting evidence that many natural products target PI3K/AKT/mTOR-mediated autophagy to treat diseases [12,13]. The objective of our study was to examine the impact of PI3K protein inhibitors on cisplatin-induced AKI.

*Flammulina velutipes* has a pleasant taste and is high in nutritional value. The substrates and fungal mycelium of *F. velutipes* contain bioactive polysaccharides, which have beneficial immunomodulatory, antioxidant, and anti-inflammatory properties [14]. This suggests that *F. velutipes* has great potential in both the food and pharmaceutical fields. *F. velutipes* polysaccharides were reported to help with antioxidative stress and provide renal protection in mice with streptozotocin-induced diabetic nephropathy [15]. The strain of *F. velutipes* employed in this work was created by crossing white and yellow strains, and its effect on nephrotoxic drug-induced AKI has not been examined.

## 2. Results

### 2.1. WFV Ameliorates Renal Changes and Renal Activity in Mice with Cisplatin-Induced AKI

**Figure 1:**
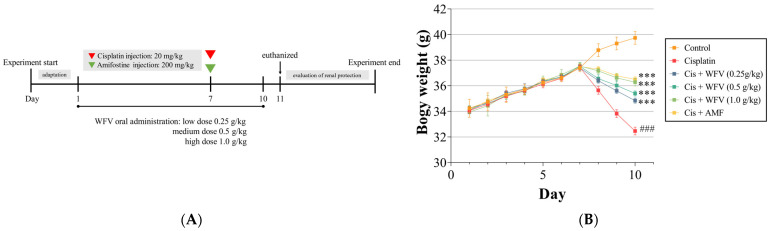

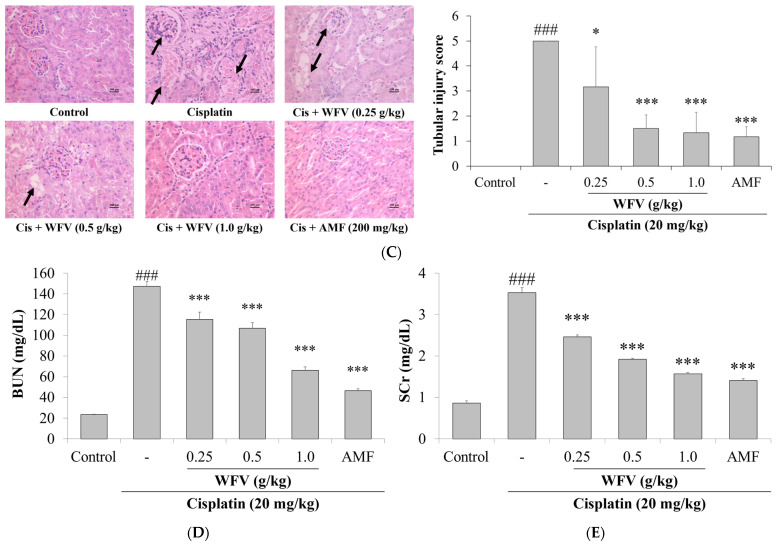
Animal experiments were used to investigate nephroprotective effect of WFV under cisplatin induction. (**A**) Procedure of experimental design. (**B**) Weight change of each group during experiment. (**C**) Histological changes of kidney and scoring of kidney damage. Histopathological analysis of kidney tissues used H&E staining and was observed under microscope (400×). Black arrows indicate renal tubular necrosis, hyaline casts, and cellular debris. Graphs show (**D**) serum BUN levels and (**E**) SCr levels. Data are expressed as mean ± SD (*n* = 5). AMF, amifostine; BUN, blood urea nitrogen; SCr, creatinine. ^###^ *p* < 0.001 compared to control group; * *p* < 0.05 and *** *p* < 0.001 compared to cisplatin group.

The experiment was designed as shown in Figure 1A. It was observed that the group of mice induced with cisplatin on the seventh day showed a decrease in bodyweight (Figure 1B), which initially indicated the success of cisplatin induction. Amifostine (AMF) is a cytoprotective agent used in cancer therapy to reduce side effects [16]. In the group given AMF, bodyweight did not significantly decrease after cisplatin injection compared to the cisplatin group. On the other hand, in the group given WFV, weight loss decreased with increasing dosages and was most pronounced at 1.0 g/kg WFV.

Acute inflammation causes swelling of the kidney, resulting in increased kidney weight [17]. Our results showed that the decreased kidney index in the group receiving WFV was positively correlated with an increased WFV dose (Table 1). The values in the 1.0 g/kg WFV + cisplatin group were noticeably different from those in the cisplatin group and resembled those in the AMF + cisplatin group.

To further observe the effect of cisplatin on renal morphology, we used the H&E staining method to stain and score the kidney sections, and the results are shown in Figure 1C. Structural integrity in the control group was preserved. In contrast, the cisplatin group presented obvious necrosis of the renal filopodia and tubular cells, and the structure of the cells was obviously loose and not dense.

The measurement of blood urea nitrogen (BUN) and creatinine (SCr) revealed renal insufficiency in the cisplatin group compared to the control group (Figure 1D,E). If WFV was given, the levels decreased with increasing WFV doses, similarly to the results of the AMF + cisplatin group.

### 2.2. WFV Restores Renal Antioxidant Capacity and Reduces Levels of Oxidative Stress in Cisplatin-Related Aki

**Figure 2:**
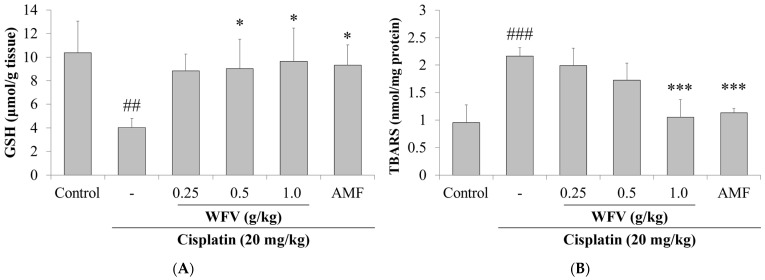
Expression of antioxidant substances and lipid peroxides in kidneys of each group was observed: (**A**) GSH levels and (**B**) MDA levels. Data are expressed as mean ± SD (*n* = 5). GSH, glutathione; MDA, malondialdehyde; TBARS, thiobarbituric acid reactive substances. ^##^ *p* < 0.01 and ^###^ *p* < 0.001 compared to control group; * *p* < 0.05 and *** *p* < 0.001 compared to cisplatin group.

**Figure 3:**
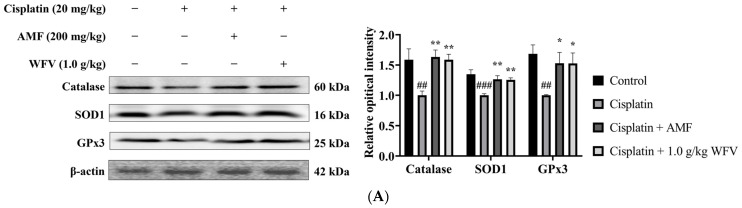

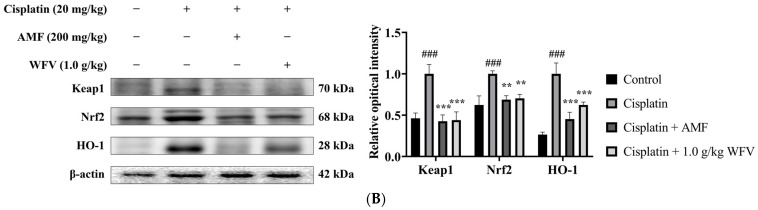
WFV alleviates oxidative stress generated by cisplatin induction. Protein expression of (**A**) antioxidant enzymes, including catalase, SOD1, and GPx3, and (**B**) Keap1, Nrf2, and HO-1 were determined by Western blot. Experiment was repeated three times, from which one set of data was selected for illustration. ^##^ *p* < 0.01 and ^###^ *p* < 0.001 compared to control group; * *p* < 0.05, ** *p* < 0.01, and *** *p* < 0.001 compared to cisplatin group.

Cisplatin decreases antioxidant enzyme activity and depletes intracellular glutathione (GSH) concentrations, leading to an accumulation of ROS in RTECs [18]. In contrast to the control group, the cisplatin group had decreased GSH levels and increased malondialdehyde (MDA) levels, as seen in Figure 2. On the other hand, by lowering the level of MDA and raising the level of GSH, the 1.0 g/kg WFV + cisplatin group had decreased oxidative stress in cisplatin-induced AKI. The protein expression levels of antioxidants in the kidney were further examined. The results show that catalase, SOD1, and GPx3 were attenuated in the cisplatin group (Figure 3A), while this condition was restored in the 1.0 g/kg WFV + cisplatin group, like in the AMF + cisplatin group.

In addition, ROS accumulation in the kidney activates Nrf2-dependent cytoprotective gene and transporter production in RTECs [19]. Figure 3B shows the expression of the Keap1/Nrf2/HO-1 pathway in the kidney. Because of the increased renal oxidative stress induced by cisplatin, the cisplatin group showed a higher expression of Keap1, Nrf2, and HO-1 than the control group. Pretreatment with WFV protects the kidney from the oxidative stress caused by ROS accumulation.

### 2.3. WFV Reduces the Renal Inflammatory Response Caused by Cisplatin Administration

**Figure 4:**
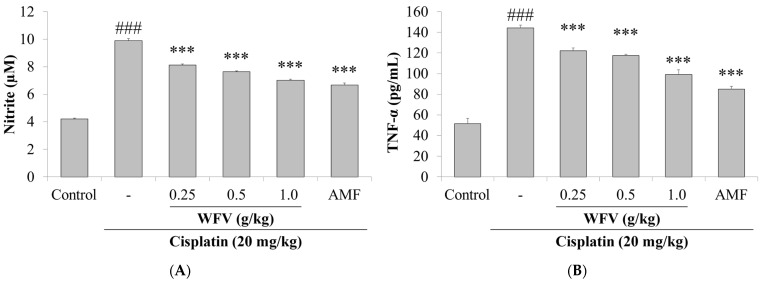

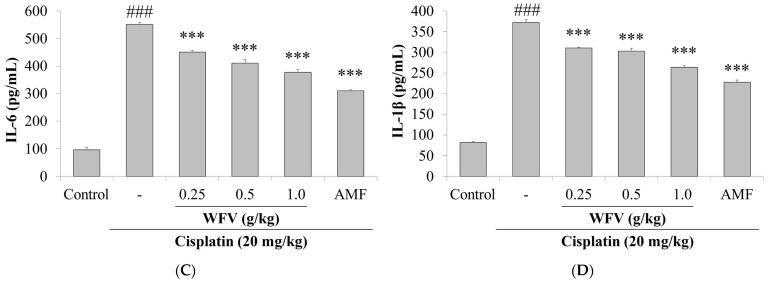
Inflammatory response of kidney was observed by measuring nitrite and inflammatory cytokines, including levels of (**A**) NO, (**B**) TNF-α, (**C**) IL-1β, and (**D**) IL-6. Data are expressed as mean ± SD (*n* = 5). ^###^ *p* < 0.001 compared to control group; *** *p* < 0.001 compared to cisplatin group.

One of the main mechanisms of cisplatin-induced nephrotoxicity is the upregulation of an inflammatory response [20]. We measured nitric oxide (NO) and inflammatory cytokines in serum to observe kidney inflammation. The results are shown in Figure 4. The concentration in the 1.0 g/kg WFV + cisplatin group was decreased compared to the cisplatin group, suggesting that WFV has anti-inflammatory effects.

**Figure 5:**
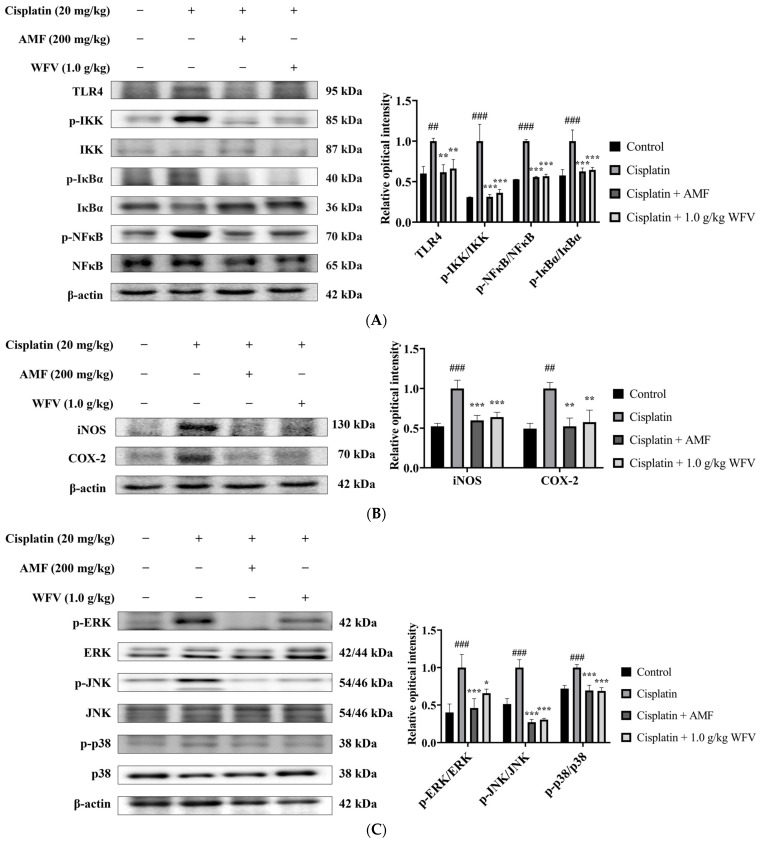
WFV ameliorates inflammatory response caused by cisplatin-induced AKI. Measurements of (**A**) TLR4/NFκB pathway correlation, including protein levels of TLR4, p-IKK/IKK, p-NFκB/NFκB, and p-IκBα/IκBα; (**B**) protein levels of iNOS and COX-2; and (**C**) MAPK pathway correlation, including protein levels of p-ERK/ERK, p-JNK/JNK, and p-38/p38. Experiment was repeated three times, from which one set of data was selected for illustration. ^##^ *p* < 0.01 and ^###^ *p* < 0.001 compared to control group; * *p* < 0.05, ** *p* < 0.01, and *** *p* < 0.001 compared to cisplatin group.

To further understand the anti-inflammatory mechanism of WFV, we tested the inflammation-related pathways using the Western blot method. Toll-like receptors (TLRs) are widely expressed in RTECs and regulate the immune response. As TLR4 is the most widely expressed TLR, its activation may lead to kidney injury [21]. The results show that the 1.0 g/kg WFV in the WFV + cisplatin group contributed to the downregulation of TLR4 compared to the cisplatin group (Figure 5A). As a downstream signal of TLR4, IKK responds to various cellular stimuli. When IKK is activated, it phosphorylates and degrades IκBα and then releases NF-κB, which is thought to be an important pathway for inflammation [22]. The results show that WFV helped to reduce the phosphorylation levels of IKK, IκBα, and NF-κB in the kidney (Figure 5A). Thus, by modulating the TLR4/NF-κB pathway in cisplatin-induced AKI, WFV contributes to downregulating inflammation.

iNOS and COX-2, the target genes of NF-kB, are pro-inflammatory cytokines [23]. As the results in Figure 5B show, similarly to the results of inflammatory cytokines in serum, iNOS and COX-2 in the kidney were highly expressed in the cisplatin group, while WFV downregulated their expression. The MAPK pathway is another downstream mediator of TLR4 signaling, and its activation has been associated with tissue damage in a variety of organs, including the kidney [24]. The phosphorylation states of MAPKs can be seen in Figure 5C. By administering WFV, the expression of p-ERK, p-JNK, and p-p38 was downregulated. Once more, these findings indicate that WFV reduced inflammation by altering the aforementioned mechanisms.

### 2.4. WFV Attenuates Cisplatin-Induced Apoptosis, Autophagy, and PI3K/AKT Pathway Expression

**Figure 6:**
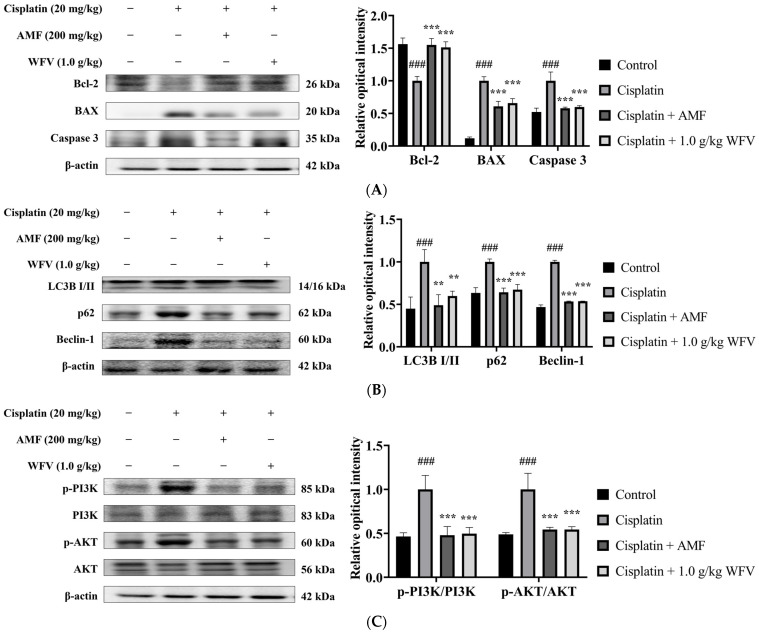
WFV downregulates increased apoptosis, autophagy, and PI3K/AKT pathway protein expression after cisplatin induction. (**A**) Apoptosis-related proteins include Bcl-2, BAX, and caspase 3. (**B**) Autophagy-related proteins were detected in LC3B, Beclin-1, and p62. (**C**) p-PI3K/PI3K and p-AKT/AKT protein expression. Experiment was repeated three times, from which one set of data was selected for illustration. ^###^ *p* < 0.001 compared to control group; ** *p* < 0.01, and *** *p* < 0.001 compared to cisplatin group.

Apoptosis is thought to be one of the mechanisms responsible for nephrotoxic cell death [25]. It was found that cisplatin activates the caspase pathway, leading to the apoptosis of RTECs [26]. The pro-apoptotic proteins BAX and caspase 3 and the anti-apoptotic protein Bcl-2 were all significantly different between the cisplatin group and the control group, as shown in Figure 6A, while their level of expression in the 1.0 g/kg WFV + cisplatin group tended to be similar to that of the AMF + cisplatin and control groups.

Autophagy, as an adaptive mechanism, is thought to be a general response to the stresses that lead to cell death [27]. It was found that the exposure of renal proximal tubular epithelial cells to cisplatin resulted in a significant increase in autophagic markers, indicating the development of autophagy [28]. As shown in Figure 6B, WFV downregulated the protein expression of LC3B I/II, p62, and Beclin-1 compared to the cisplatin group. These results suggest that the pre-administration of WFV helped to protect from cisplatin-induced nephrotoxicity and improved the expression of apoptosis and autophagy in the kidney, which is similar to the results of the AMF + cisplatin group.

Cellular stress triggers the PI3K/AKT pathway, which is then involved in the regulation of a number of physiological activities within cells [29]. It was found that AKT initiates autophagy by activating downstream signaling [30]. Our results show that the accumulation of oxidative stress caused an increase in PI3K and AKT expression in the cisplatin group (Figure 6C). The results of AMF and WFV administration were similar to the results of the control group, indicating that WFV protects the kidney from cisplatin-induced cellular stress.

### 2.5. Use of PI3K Inhibitor Blocks Subsequent Signals and Increases Kidney Damage Caused by Cisplatin

**Figure 7:**
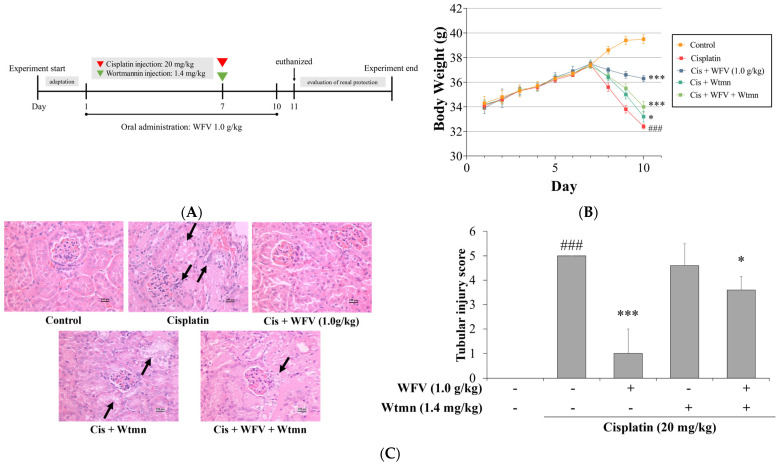

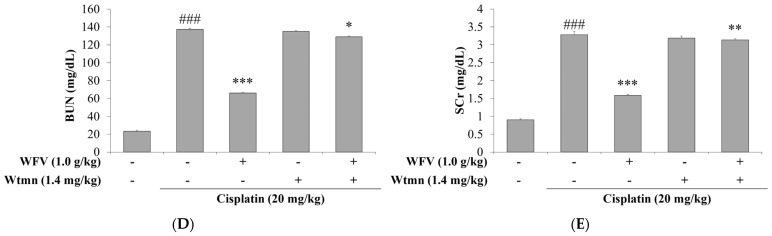
Renoprotective effects of WFV and PI3K inhibitor under cisplatin induction. (**A**) Experimental design. (**B**) Weight changes in each group during experiment. (**C**) Histological changes in kidneys and scoring of renal injury. Histopathological analysis of kidney tissues was performed using H&E staining and observed under a microscope (400×). Black arrows indicate renal tubular necrosis, hyaline casts, and cellular debris. Graphs show serum (**D**) BUN and (**E**) SCr levels. Data are expressed as mean ± SD (*n* = 5). Wtmn, Wortmannin. ^###^ *p* < 0.001 compared to control group; * *p* < 0.05, ** *p* < 0.01, and *** *p* < 0.001 compared to cisplatin group.

Wortmannin (Wtmn), a PI3K inhibitor, was further used in this experiment to observe the effect of the PI3K/AKT pathway in the cisplatin-induced AKI mouse model and further understand the protective effect of WFV. The experimental design is shown in Figure 7A. The bodyweight in each group was observed (Figure 7B). Weight loss was most pronounced in the cisplatin group after induction on day 7 and was recovered slightly in the cisplatin + Wtmn group, but there was no significant difference in the renal index between the cisplatin groups with and without Wtmn (Table 2). However, the administration of 1.0 g/kg WFV helped to improve bodyweight and renal index changes in the mice. In addition, the results of kidney sections show that the effects of cisplatin with/without Wtmn were similar, such as a less complete cytoarchitecture and tubular necrosis and more hyaline casts, which WFV helped to improve (Figure 7C). Furthermore, it can be seen in Figure 7D,E that after cisplatin induction mice administered cisplatin with/without Wtmn had higher BUN and SCr levels. Conversely, the WFV + Wtmn group had lower BUN and SCr levels.

### 2.6. Anti-Inflammatory and Anti-Oxidative Stress Responses of WFV with or without Wtmn in Cisplatin-Induced AKI

**Figure 8:**
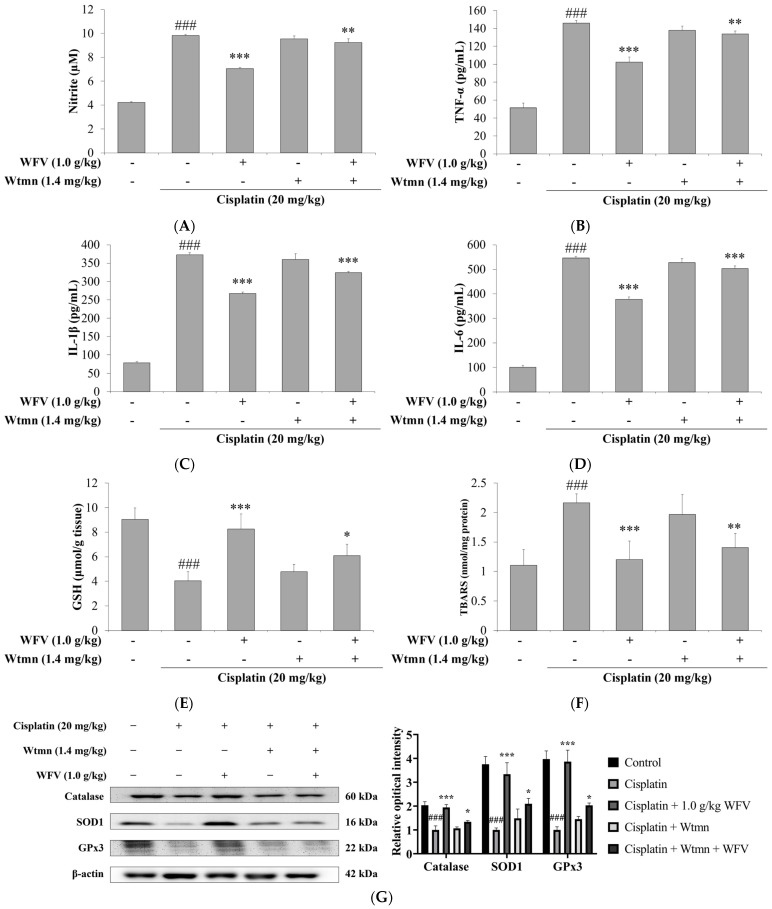
WFV with or without PI3K inhibitor influenced inflammation and oxidative stress. Measurements include levels of (**A**) NO, (**B**) TNF-α, (**C**) IL-1β, (**D**) IL-6, (**E**) GSH, and (**F**) MDA. Data are expressed as mean ± SD (*n* = 5). (**G**) Antioxidant enzymes were detected by Western blot, including catalase, SOD1, and GPx3. Experiment was repeated three times, from which one set of data was selected for illustration. ^###^ *p* < 0.001 compared to control group; * *p* < 0.05, ** *p* < 0.01, and *** *p* < 0.001 compared to cisplatin group.

To understand how PI3K inhibition by Wtmn affects the anti-inflammatory and antioxidative stress response of the kidney, we evaluated the related protein expression. As shown in Figure 8A–D, the WFV + Wtmn group had lower serum levels of NO and inflammatory cytokines compared to the Wtmn group. In terms of antioxidative stress effects, as previously tested for GSH and MDA, the results in Figure 8E,F show that the cisplatin group with/without Wtmn had lower concentrations of GSH and higher MDA, and these were improved in the WFV + Wtmn group. The expression levels of catalase, SOD1, and GPx3 antioxidant enzymes are shown in Figure 8G, and the results are similar to those of GSH, again showing that PI3K inhibition affects the performance of the kidney in an oxidative stress environment caused by cisplatin.

### 2.7. Effect of WFV with or without Wtmn on Autophagy and PI3K/AKT Protein Performance

**Figure 9:**
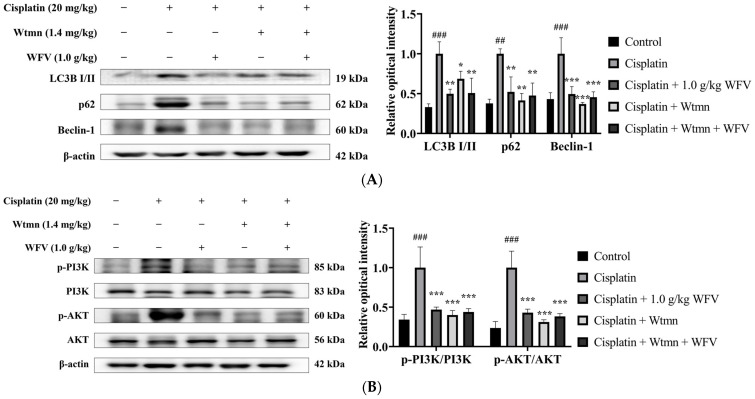
PI3K inhibitor affected autophagy and PI3K/AKT pathway performance, which could be ameliorated by WFV. Results include (**A**) LC3B, Beclin-1, and p62 protein expression and (**B**) p-PI3K/PI3K and p-AKT/AKT protein expression. Experiment was repeated three times, from which one set of data was selected for illustration. ^##^ *p* < 0.01 and ^###^ *p* < 0.001 compared to control group; * *p* < 0.05, ** *p* < 0.01, and *** *p* < 0.001 compared to cisplatin group.

The results of the effect of WFV with/without Wtmn on autophagy and PI3K/AKT protein are shown in Figure 9. Similarly to the previous results, the cisplatin group showed an increased expression of LC3B I/II, p62, and Beclin-1, while Wtmn suppressed their expression (Figure 9A). The results are similar for WFV with and without Wtmn. It is further suggested that the pretreatment of mice with 1.0 g/kg WFV can attenuate cisplatin-induced AKI by affecting autophagy production. Finally, p-PI3K and p-AKT protein expression indicated that Wtmn interfered with the PI3K/AKT pathway. The Wtmn + cisplatin group had a decreased expression compared to the cisplatin group, while the WFV + Wtmn group had a recovered protein expression (Figure 9B). The findings show once more how cisplatin-induced AKI in mice is influenced by the stability of the PI3K/AKT pathway, as well as how oral WFV has renoprotective effects on the kidney.

### 2.8. Determination of WFV by High-Performance Liquid Chromatography

**Figure 10:**
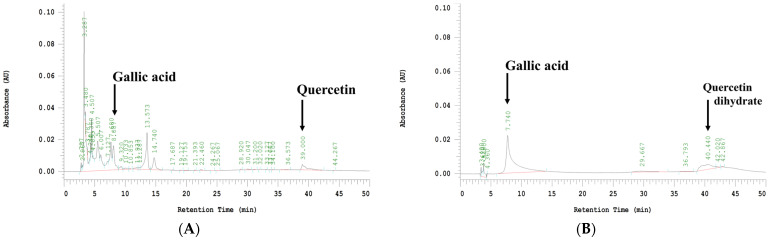
HPLC profiles of WFV and standard compounds. (**A**) Water extract from brown strain of *F. velutipes* (WFV) and (**B**) 50 ppm gallic acid plus 200 ppm quercetin dihydrate as standard.

ROS affect kidney disease, and antioxidant intervention via diet or medication can alleviate kidney damage [31]. Polyphenols have great bioactive potential to scavenge excess free radicals and help alleviate the side effects caused by cisplatin [32,33]. To confirm the potential composition of WFV, an analysis was performed using HPLC-DAD [34]. As shown in Figure 10, WFV detected gallic acid at 8.087 min with relative content measured as 25.25 μg/mL; quercetin was detected at 39.000 min with relative content measured as 144.54 μg/mL.

## 3. Discussion

Humans have consumed mushrooms for a long time, and many studies have found that they have a wide range of medicinal uses [35], including immunomodulatory [36], antioxidant [37], antiviral [38], hepatoprotective, and antidiabetic [39,40] effects. *Flammulina velutipes* is a mushroom that is consumed in large quantities, and its composition includes polysaccharides, phenols, flavonoids, and terpenes [34,41]. However, the brown strain of *F. velutipes* is relatively new and has not been extensively studied for its renal protective effects. Similarly to the way humans consume *F. velutipes*, extraction is carried out with water. Many natural products (flavonoids, terpenes, saponins, polyphenols, and polysaccharides) have been previously reported to modulate pathways associated with cisplatin-induced AKI [42,43]. In our study, WFV was found to contain gallic acid and quercetin. Previous studies have found that these two polyphenols have beneficial protective effects on the kidneys. Gallic acid improved renal oxidative stress, inflammation, and mitochondrial dysfunction in rats with cisplatin-induced acute nephrotoxicity [44,45], and quercetin significantly reduced serum SCr and BUN and inflammatory factors in mice with cisplatin-induced AKI [46].

Current studies of cisplatin-induced AKI have established that a single injection of 20 mg/kg bodyweight of cisplatin in mice can cause significant renal damage. BUN and SCr are usually used as markers of damage and observed changes in renal morphology [47]. AMF has been evaluated as a cytoprotective agent against cisplatin toxicity. However, it may also cause a range of adverse effects, including nausea, vomiting, and low blood pressure. Therefore, we used AMF as a positive control group to observe whether WFV had a similar renoprotective effect. Our results show that AMF and WFV alleviated weight loss and the renal index compared to the cisplatin group. The renal biomarker measurement showed a significant improvement in WFV-reversed BUN and SCr levels. Cisplatin induces increased infiltration of cytokines such as interleukins and neutrophils in the kidney [48]. The results of H&E sections showed that cisplatin caused severe renal damage, such as tissue vacuole formation and the infiltration of inflammatory cells. It was discovered that cisplatin-induced toxicity is linked to a reduction in the amount of GSH and protein-binding SH groups in cells, which leads to an accumulation of free radicals and oxidative stress [49]. Our findings demonstrate that, compared to the cisplatin group, the 1.0 g/kg WFV + cisplatin group had dramatically lower MDA levels and reduced GSH and antioxidant enzymes. In addition, the cisplatin + 0.5 and 1.0 g/kg WFV groups had significantly downregulated TNF-α, IL-6, and IL-1β compared to the cisplatin group; thus, the pathological sites in the sections were improved, and the results were similar to those of the AMF + cisplatin group.

ROS accumulation in the kidney activates Nrf2 in RTECs [19] and increases the expression of the oxidative stress marker HO-1 [50]. In the current study, the pretreatment of mice with 1.0 g/kg WFV led to the downregulation of the Keap1/Nrf2/HO-1 pathway in the kidney. An ROS acts as a central regulator of inflammatory signaling, activating inflammatory signals such as NF-κB [51]. According to a study, *F. velutipes* water extract can reduce the severity of inflammatory bowel illness caused by dextran sulfate sodium by altering the TLR4/NF-κB pathway [52]. Our findings suggest that WFV may shield the kidney from cisplatin-induced inflammatory reactions by inhibiting TLR4 and IKK, Iκβα, and NF-κB phosphorylation and lowering iNOS and COX2 protein production. The MAPK pathway transmits signals from a variety of stimuli and elicits appropriate physiological responses, including cell proliferation, inflammatory responses, and apoptosis [53]. It is another important signal downstream of TLR4, and cisplatin activates p38, ERK, and JNK/SAPK in RTECs [54]. In the present study, MAPK pathway expression was increased in the cisplatin group, while the 1.0 g/kg WFV + cisplatin group was similar to the AMF group and trended towards the control group.

The underlying mechanism of cisplatin-induced AKI involves programmed death in addition to oxidative stress and inflammation. Reducing apoptosis and autophagy may reduce renal inflammation [47]. The Bcl-2 and caspase families regulate apoptotic pathways [55]. In addition to the MAPK pathway, WFV can downregulate BAX and caspase 3 expression and upregulate Bcl-2 expression, thereby reducing cisplatin-induced apoptosis. In the current study, WFV ameliorated cisplatin-induced autophagic overexpression by downregulating LC3, p62, and Beclin-1 protein expression. PI3K is an enzyme that modulates many physiological functions by phosphorylating phospholipids, and Beclin-1 modulates autophagy by forming complexes with class III PI3K [56]. Additionally, evidence from recent studies suggests that carvacrol reduces cisplatin-induced AKI by modifying the PI3K/AKT pathway [57]. In the present investigation, we found that cisplatin treatment resulted in the activation of the kidney PI3K/AKT pathway. Further, we demonstrated that the use of PI3K inhibitor caused the inhibition of autophagy and did not help to alleviate cisplatin-induced AKI. The cisplatin + Wtmn group showed similar severe results to the cisplatin group, such as the results for tubular injury score, BUN, Scr, and the renal index. The inhibition of PI3K made the results of GSH and MDA of the WFV group worse than the group without Wtmn. Finally, it was demonstrated that treatment with WFV can regulate the cisplatin-induced activation of the PI3K/AKT pathway and autophagy.

## 4. Materials and Methods

### 4.1. Sample Preparation

The brown strain of *F. velutipes* was obtained from Wantchange Agricultural Biotechnology Co., Ltd. (Taichung, Taiwan). The brown strain of *F. velutipes* and water in a ratio of 1:5 (*w*/*w*) were heated to 100 °C for 60 min. The liquid was collected, filtered, and the filtered extract was freeze-dried. The extraction rate is 4.45%. This extract is called water extract from the brown strain of *F. velutipes* (WFV). Before the experiment, WFV was prepared in reverse osmosis water to concentrations of 0.25, 0.5, and 1.0 g/kg for animal experiments.

### 4.2. Chemicals and Reagents

Gallic acid, quercetin dihydrate, cisplatin, amifostine, Wortmannin, and other reagents were obtained from Sigma-Aldrich (St. Louis, MO, USA). Blood urea nitrogen and creatinine test kits were purchased from HUMAN Diagnostics Worldwide (Wiesbaden, Germany). ELISA kits for cytokines TNF-α, IL-1β, and IL-6 were obtained from BioLegend (San Diego, CA, USA). Antibodies against AKT, p-AKT, caspase 3, inducible NO synthase (iNOS), cyclooxygenase-2 (COX-2), catalase, glutathione peroxidase 3 (GPx3), Toll-like receptor 4 (TLR-4), NF-κB, p-NF-κB, IκBα, p-IκBα, p-ERK, and p-JNK were purchased from GeneTex (Irvine, CA, USA). Antibodies against Bcl-2, JNK, LC3B, Beclin-1, and p62 were purchased from Cell Signaling (Danvers, MA, USA). Antibodies against superoxide dismutase 1 (SOD1) were purchased from BioVision (Milpitas, CA, USA). Antibodies against p-PI3K were purchased from Elabscience (Houston, TX, USA). Antibodies against PI3K, ERK, IKK, and p-IKK were purchased from Invitrogen (Waltham, MA, USA). Antibodies against BAX, Keap1, Nrf2, heme oxygenase-1 (HO-1), p38, p-p38, and β-actin were obtained from Abcam (Cambridge, UK). β-actin was used as an internal control.

### 4.3. Mouse Model and Research Design

Male ICR mice (8 weeks old, weighing 30–35 g) were purchased from BioLASCO Taiwan Co., Ltd. (Taipei, Taiwan). Animals were maintained according to the standard animal protocol approved by China Medical University (CMUIACUC-2019-377). Starting 3 days before the experiment, mice were subjected to a 12 h light/dark cycle, with temperature maintained at 23 °C and relative humidity at 50%. Mice were divided into six groups (5 mice per group): (1) control group; (2) cisplatin group; (3) AMF + cisplatin group; (4) low-dose (0.25 g/kg) WFV + cisplatin group; (5) medium-dose (0.5 g/kg) WFV + cisplatin group; (6) high-dose (1.0 g/kg) WFV + cisplatin group. AMF was used as positive control. Based on previous results, mice were orally administered WFV at doses of 0.25, 0.5, or 1.0 g/kg once daily for 10 days [58]. The dose of cisplatin was selected based on a previous study [47]. Mice were given a single intraperitoneal (IP) injection of 20 mg/kg cisplatin dissolved in 0.5% carboxymethyl cellulose solution on day 7 to induce AKI. Mice were sacrificed 72 h after cisplatin injection, and blood samples were collected to measure serum biomarkers. One kidney was collected for tissue sectioning, and the other kidney was collected for subsequent analysis.

In order to understand how Wtmn affects cisplatin-induced AKI, another batch of animal experiments was conducted in which mice were divided into five groups (5 mice per group): (1) control group; (2) cisplatin group; (3) cisplatin + Wtmn group; (4) cisplatin + WFV group; (5) cisplatin + WFV + Wtmn group. Mice were given 1.0 g/kg WFV orally for 10 days. Mice given cisplatin received a single IP injection of 20 mg/kg on day 7; mice given Wtmn received an IP injection of 1.4 mg/kg 30 min before cisplatin administration [59]. Serum and kidneys were processed as described above.

### 4.4. Bodyweight and Kidney Index

Between the start of the experiment and the day of sacrifice, each mouse’s bodyweight was recorded. Kidneys were immediately dissected and collected after sacrifice. The kidneys were weighed, and the kidney index (kidney weight/bodyweight (mg/g)) was calculated.

### 4.5. Histological Tests

Kidney tissue sections were stained with hematoxylin and eosin (H&E) for microscopic examination (Nikon Eclipse TS100, Tokyo, Japan). Depending on the degree, kidney damage was divided into six grades from 1 to 5: <1% minimal; 1–25% slight; 26–50% moderate; 51–75% moderate/severe; >75% severe/high [60].

### 4.6. Renal Biomarker Measurements

After the serum of each group was collected, urea nitrogen levels and serum creatinine were measured using an automated biochemical analyzer (Roche Diagnostics, Cobas Mira Plus, Mannheim, Germany) to analyze the changes in renal function in mice.

### 4.7. Serum Nitric Oxide Level Measurement

The Griess reaction colorimetric method was used to quantify serum NO levels, with the culture supernatant and Griess reagent mixed in equal proportions. After incubation at 540 nm for 10 min, the absorbance was measured utilizing a microplate reader [61] (Molecular Devices, Orleans Drive, Sunnyvale, CA, USA).

### 4.8. Lipid Peroxidation Assay

The evaluation of TBARS (thiobarbituric acid reactive substance) was performed by measuring the levels of malondialdehyde (MDA) arising from renal lipid peroxidation [62]. To prepare the extracts, kidneys were lysed using a lysis buffer on ice. Thiobarbituric acid (TBA) solution was added and incubated with the extracts at 90 °C for 45 min to form the MDA–TBA adduct. The amount of TBARS formed was then measured at 532 nm against blank using a microplate reader (Molecular Devices, Orleans Drive, Sunnyvale, CA, USA).

### 4.9. Glutathione Assay

The homogenization of tissues was performed using trichloroacetic acid (TCA) buffer to acquire a supernatant. Next, 5,5’-dithio-bis-(2-nitrobenzoic acid) (DTNB) was added and the resulting mixture was left to stand for 10 min. Measuring the absorbance at 412 nm allowed for determination of the concentration, which was then compared to a standard curve that had been generated using a known quantity of GSH [63].

### 4.10. Cytokine Assay

For evaluation of the inflammatory activity, the serum levels of TNF-α, IL-1β, and IL-6 were examined with commercially available ELISA kits according to the manufacturer’s recommended protocol (BioLegend, San Diego, CA, USA).

### 4.11. Western Blot Analysis

The tissues were extracted and collected following treatment with the designated drug concentration for 24 h. The extraction buffer, RIPA buffer, was purchased from GENESTAR (Kaohsiung, Taiwan), and the concentration of total protein was measured using a Bio-Rad protein assay kit (BioRad, Hercules, CA, USA). The proteins (20 μg/well) were separated by gel electrophoresis and transferred to a membrane. A series of treatments, including primary and secondary antibodies, horseradish peroxidase (HRP) conjugate, and ECL substrate, were applied to detect signals, which were identified using Kodak Gel Logic 1500 Imaging System (Eastman Kodak Company, Rochester, NY, USA) [64].

### 4.12. High-Performance Liquid Chromatography (HPLC) Analysis

After modifying the conditions based on a previous study [34], the polyphenol content of WFV was analyzed by HPLC (Hitachi Ltd., Tokyo, Japan). A 250 × 4.6 mm i.d. 5 μm reversed-phase TSKgel Tosoh ODS-80Tm chromatographic column (Tosoh, Yamaguchi, Japan) and a diode array detector (DAD) were used for the analysis. The mobile phases were acetonitrile (A) and 0.5% acetic acid (B). The gradient elution procedure for the mobile phases was as follows: 0–20 min: 2–10% A and 98–90% B; 20–30 min: 10–25% A and 90–75% B; 30–40 min: 25–35% A and 75–65% B; 40–50 min: 35–2% A and 65–98% B. The procedure used a flow rate of 1.0 mL/min. The wavelength and column temperatures were found to be 280 nm and 35 °C, respectively.

### 4.13. Statistical Analysis

Statistical analysis was performed using SPSS software 22.0 (SPSS, Inc., Chicago, IL, USA). Data were analyzed using one-way analysis of variance (ANOVA), followed by Scheffe’ test, and data were expressed as mean ± standard deviation (SD). *p*-values and significance levels are indicated by ticks or asterisks (^#^, *) with significance levels <0.05, <0.01, and <0.001.

## 5. Conclusions

**Figure 11:**
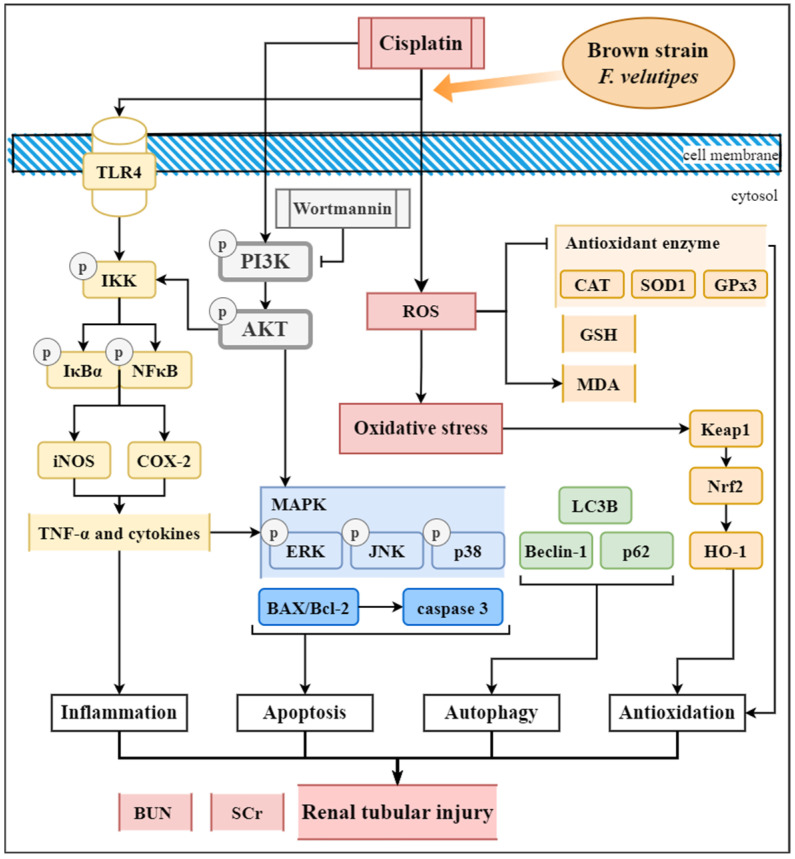
Mechanism of experimental results in this paper. Effects of cisplatin-induced acute kidney injury in mice were observed in pathways related to oxidative stress, inflammation, apoptosis, and autophagy. (Drawn by draw.io.)

The experimental mechanism is illustrated in Figure 11. This study demonstrated the following: Pretreatment with oral WFV reduced cisplatin-induced kidney injury, as assessed based on renal function and histology. WFV demonstrated antioxidant, anti-inflammatory, and anti-apoptotic functions in cisplatin-induced AKI. WFV administration significantly reduced autophagy activation. With the use of PI3K inhibitors, the protective effect of WFV on the kidneys was found to be associated with the PI3K/AKT pathway. Overall, our study confirms the pharmacological activity of the brown strain of *F. velutipes* for renal protection via the TLR4/NF-κB, MAPK, Nrf2/HO-1, and PI3K/AKT pathways and provides new evidence for its use as a nutritional support for patients with AKI.

## Figures and Tables

**Table 1:** Renal weight and kidney index for each group.

Groups	Dosage (g/kg)	Renal Weight (g)	Kidney Index (mg/g)
Control	-	0.54 ± 0.01	1.36 ± 0.06
Cisplatin	0.02	0.75 ± 0.02 ^###^	2.31 ± 0.07 ^###^
Cisplatin + WFV	0.25	0.67 ± 0.01 ***	1.92 ± 0.04 ***
Cisplatin + WFV	0.5	0.65 ± 0.01 ***	1.86 ± 0.04 ***
Cisplatin + WFV	1.0	0.61 ± 0.01 ***	1.68 ± 0.03 ***
Cisplatin + AMF	0.2	0.55 ± 0.02 ***	1.50 ± 0.07 ***

Data are expressed as mean ± SD (*n* = 5). ^###^ *p* < 0.001 compared to control group; *** *p* < 0.001 compared to cisplatin group.

**Table 2:** Use of WFV with or without PI3K inhibitor altered kidney index. Data are expressed as mean ± SD (*n* = 5).

Group	Renal Weight (g)	Kidney Index (mg/g)
Control	0.52 ± 0.03	1.33 ± 0.08
Cisplatin	0.75 ± 0.03 ^###^	2.33 ± 0.10 ^###^
Cisplatin + WFV (1.0 g/kg)	0.61 ± 0.01 ***	1.68 ± 0.03 ***
Cisplatin + Wtmn (1.4 mg/kg)	0.75 ± 0.02	2.27 ± 0.04
Cisplatin + WFV + Wtmn	0.71 ± 0.01	2.11 ± 0.01 **

^###^ *p* < 0.001 compared to control group; ** *p* < 0.01 and *** *p* < 0.001 compared to cisplatin group.

## Data Availability

The data are contained within the article.

## References

[1] Lazzareschi D., Mehta R.L., Dember L.M., Bernholz J., Turan A., Sharma A., Kheterpal S., Parikh C.R., Ali O., Schulman I.H. (2023). Overcoming Barriers in the Design and Implementation of Clinical Trials for Acute Kidney Injury: A Report from the 2020 Kidney Disease Clinical Trialists Meeting. Nephrol. Dial. Transplant..

[2] Kellum J.A., Romagnani P., Ashuntantang G., Ronco C., Zarbock A., Anders H.-J. (2021). Acute Kidney Injury. Nat. Rev. Dis. Prim..

[3] Thongprayoon C., Hansrivijit P., Kovvuru K., Kanduri S.R., Torres-Ortiz A., Acharya P., Gonzalez-Suarez M.L., Kaewput W., Bathini T., Cheungpasitporn W. (2020). Diagnostics, Risk Factors, Treatment and Outcomes of Acute Kidney Injury in a New Paradigm. J. Clin. Med..

[4] Perazella M.A., Rosner M.H. (2022). Drug-Induced Acute Kidney Injury. CJASN.

[5] Ali R., Aouida M., Alhaj Sulaiman A., Madhusudan S., Ramotar D. (2022). Can Cisplatin Therapy Be Improved? Pathways that Can Be Targeted. Int. J. Mol. Sci..

[6] Ranasinghe R., Mathai M.L., Zulli A. (2022). Cisplatin for Cancer Therapy and Overcoming Chemoresistance. Heliyon.

[7] Soni H., Kaminski D., Gangaraju R., Adebiyi A. (2018). Cisplatin-Induced Oxidative Stress Stimulates Renal Fas Ligand Shedding. Ren. Fail..

[8] Mercantepe F., Mercantepe T., Topcu A., Yılmaz A., Tumkaya L. (2018). Protective Effects of Amifostine, Curcumin, and Melatonin against Cisplatin-Induced Acute Kidney Injury. Naunyn-Schmiedeberg’s Arch. Pharm..

[9] Alassaf N., Attia H. (2023). Autophagy and Necroptosis in Cisplatin-Induced Acute Kidney Injury: Recent Advances Regarding Their Role and Therapeutic Potential. Front. Pharmacol..

[10] Karwasra R., Kalra P., Gupta Y.K., Saini D., Kumar A., Singh S. (2016). Antioxidant and Anti-Inflammatory Potential of Pomegranate Rind Extract to Ameliorate Cisplatin-Induced Acute Kidney Injury. Food Funct..

[11] Deng J.-S., Jiang W.-P., Chen C.-C., Lee L.-Y., Li P.-Y., Huang W.-C., Liao J.-C., Chen H.-Y., Huang S.-S., Huang G.-J. (2020). *Cordyceps cicadae* Mycelia Ameliorate Cisplatin-Induced Acute Kidney Injury by Suppressing the TLR4/NF-*κ* B/MAPK and Activating the HO-1/Nrf2 and Sirt-1/AMPK Pathways in Mice. Oxidative Med. Cell. Longev..

[12] Wu X., Liu Z., Yu X., Xu S., Luo J. (2021). Autophagy and Cardiac Diseases: Therapeutic Potential of Natural Products. Med. Res. Rev..

[13] Xu Z., Han X., Ou D., Liu T., Li Z., Jiang G., Liu J., Zhang J. (2020). Targeting PI3K/AKT/MTOR-Mediated Autophagy for Tumor Therapy. Appl. Microbiol. Biotechnol..

[14] Tang C., Hoo P.C.-X., Tan L.T.-H., Pusparajah P., Khan T.M., Lee L.-H., Goh B.-H., Chan K.-G. (2016). Golden Needle Mushroom: A Culinary Medicine with Evidenced-Based Biological Activities and Health Promoting Properties. Front. Pharmacol..

[15] Lin L., Cui F., Zhang J., Gao X., Zhou M., Xu N., Zhao H., Liu M., Zhang C., Jia L. (2016). Antioxidative and Renoprotective Effects of Residue Polysaccharides from Flammulina Velutipes. Carbohydr. Polym..

[16] Kemp G., Rose P., Lurain J., Berman M., Manetta A., Roullet B., Homesley H., Belpomme D., Glick J. (1996). Amifostine Pretreatment for Protection against Cyclophosphamide-Induced and Cisplatin-Induced Toxicities: Results of a Randomized Control Trial in Patients with Advanced Ovarian Cancer. JCO.

[17] Ali B.H., Ramkumar A., Madanagopal T.T., Waly M.I., Tageldin M., Al-Abri S., Fahim M., Yasin J., Nemmar A. (2014). Motor and Behavioral Changes in Mice with Cisplatin-Induced Acute Renal Failure. Physiol. Res..

[18] Ozbek E. (2012). Induction of Oxidative Stress in Kidney. Int. J. Nephrol..

[19] Clerici S., Boletta A. (2020). Role of the KEAP1-NRF2 Axis in Renal Cell Carcinoma. Cancers.

[20] McSweeney K.R., Gadanec L.K., Qaradakhi T., Ali B.A., Zulli A., Apostolopoulos V. (2021). Mechanisms of Cisplatin-Induced Acute Kidney Injury: Pathological Mechanisms, Pharmacological Interventions, and Genetic Mitigations. Cancers.

[21] Zhang B., Ramesh G., Uematsu S., Akira S., Reeves W.B. (2008). TLR4 Signaling Mediates Inflammation and Tissue Injury in Nephrotoxicity. JASN.

[22] Zhang H., Sun S.-C. (2015). NF-ΚB in Inflammation and Renal Diseases. Cell Biosci..

[23] Yamamoto Y., Gaynor R.B. (2004). IκB Kinases: Key Regulators of the NF-ΚB Pathway. Trends Biochem. Sci..

[24] West A.P., Koblansky A.A., Ghosh S. (2006). Recognition and Signaling by Toll-Like Receptors. Annu. Rev. Cell Dev. Biol..

[25] Lau A.H. (1999). Apoptosis Induced by Cisplatin Nephrotoxic Injury. Kidney Int..

[26] Kaushal G.P., Kaushal V., Hong X., Shah S.V. (2001). Role and Regulation of Activation of Caspases in Cisplatin-Induced Injury to Renal Tubular Epithelial Cells. Kidney Int..

[27] Volarevic V., Djokovic B., Jankovic M.G., Harrell C.R., Fellabaum C., Djonov V., Arsenijevic N. (2019). Molecular Mechanisms of Cisplatin-Induced Nephrotoxicity: A Balance on the Knife Edge between Renoprotection and Tumor Toxicity. J. Biomed. Sci..

[28] Periyasamy-Thandavan S., Jiang M., Wei Q., Smith R., Yin X.-M., Dong Z. (2008). Autophagy Is Cytoprotective during Cisplatin Injury of Renal Proximal Tubular Cells. Kidney Int..

[29] Rodon J., Dienstmann R., Serra V., Tabernero J. (2013). Development of PI3K Inhibitors: Lessons Learned from Early Clinical Trials. Nat. Rev. Clin. Oncol..

[30] Kma L., Baruah T.J. (2022). The Interplay of ROS and the PI3K/Akt Pathway in Autophagy Regulation. Biotechnol. Appl. Biochem..

[31] Guerreiro Í., Ferreira-Pêgo C., Carregosa D., Santos C.N., Menezes R., Fernandes A.S., Costa J.G. (2022). Polyphenols and Their Metabolites in Renal Diseases: An Overview. Foods.

[32] Su Y.-C., Huang G.-J., Lin J.-G. (2022). Chinese Herbal Prescriptions for COVID-19 Management: Special Reference to Taiwan Chingguan Yihau (NRICM101). Front. Pharmacol..

[33] Chien L.-H., Wu C.-T., Deng J.-S., Jiang W.-P., Huang W.-C., Huang G.-J. (2021). Salvianolic Acid C Protects against Cisplatin-Induced Acute Kidney Injury through Attenuation of Inflammation, Oxidative Stress and Apoptotic Effects and Activation of the CaMKK–AMPK–Sirt1-Associated Signaling Pathway in Mouse Models. Antioxidants.

[34] Ma S., Zhang H., Xu J. (2021). Characterization, Antioxidant and Anti-Inflammation Capacities of Fermented Flammulina Velutipes Polyphenols. Molecules.

[35] Wasser S.P. (2011). Current Findings, Future Trends, and Unsolved Problems in Studies of Medicinal Mushrooms. Appl. Microbiol. Biotechnol..

[36] Zhao S., Gao Q., Rong C., Wang S., Zhao Z., Liu Y., Xu J. (2020). Immunomodulatory Effects of Edible and Medicinal Mushrooms and Their Bioactive Immunoregulatory Products. J. Fungi.

[37] Mau J.-L., Lin H.-C., Chen C.-C. (2002). Antioxidant Properties of Several Medicinal Mushrooms. J. Agric. Food Chem..

[38] Teplyakova T.V., Kosogova T.A. (2016). Antiviral Effect of Agaricomycetes Mushrooms (Review). Int. J. Med. Mushrooms.

[39] Martel J., Ojcius D.M., Chang C.-J., Lin C.-S., Lu C.-C., Ko Y.-F., Tseng S.-F., Lai H.-C., Young J.D. (2017). Anti-Obesogenic and Antidiabetic Effects of Plants and Mushrooms. Nat. Rev. Endocrinol..

[40] Soares A., de Sá-Nakanishi A., Bracht A., da Costa S., Koehnlein E., de Souza C., Peralta R. (2013). Hepatoprotective Effects of Mushrooms. Molecules.

[41] Hu Q., Yu J., Yang W., Kimatu B.M., Fang Y., Ma N., Pei F. (2016). Identification of Flavonoids from Flammulina Velutipes and Its Neuroprotective Effect on Pheochromocytoma-12 Cells. Food Chem..

[42] Fang C.-Y., Lou D.-Y., Zhou L.-Q., Wang J.-C., Yang B., He Q.-J., Wang J.-J., Weng Q.-J. (2021). Natural Products: Potential Treatments for Cisplatin-Induced Nephrotoxicity. Acta Pharm. Sin..

[43] Kang H.G., Lee H.K., Cho K.B., Park S.I. (2021). A Review of Natural Products for Prevention of Acute Kidney Injury. Medicina.

[44] Akomolafe S.F., Akinyemi A.J., Anadozie S.O. (2014). Phenolic Acids (Gallic and Tannic Acids) Modulate Antioxidant Status and Cisplatin Induced Nephrotoxicity in Rats. Int. Sch. Res. Not..

[45] Dehghani M.A., Shakiba Maram N., Moghimipour E., Khorsandi L., Atefi khah M., Mahdavinia M. (2020). Protective Effect of Gallic Acid and Gallic Acid-Loaded Eudragit-RS 100 Nanoparticles on Cisplatin-Induced Mitochondrial Dysfunction and Inflammation in Rat Kidney. Biochim. Biophys. Acta (BBA)-Mol. Basis Dis..

[46] Tan R., Wang C., Deng C., Zhong X., Yan Y., Luo Y., Lan H., He T., Wang L. (2020). Quercetin Protects against Cisplatin-induced Acute Kidney Injury by Inhibiting Mincle/Syk/NF-κB Signaling Maintained Macrophage Inflammation. Phytother. Res..

[47] Holditch S.J., Brown C.N., Lombardi A.M., Nguyen K.N., Edelstein C.L. (2019). Recent Advances in Models, Mechanisms, Biomarkers, and Interventions in Cisplatin-Induced Acute Kidney Injury. Int. J. Mol. Sci..

[48] Faubel S., Lewis E.C., Reznikov L., Ljubanovic D., Hoke T.S., Somerset H., Oh D.-J., Lu L., Klein C.L., Dinarello C.A. (2007). Cisplatin-Induced Acute Renal Failure Is Associated with an Increase in the Cytokines Interleukin (IL)-1β, IL-18, IL-6, and Neutrophil Infiltration in the Kidney. J. Pharm. Exp..

[49] Dasari S., Bernard Tchounwou P. (2014). Cisplatin in Cancer Therapy: Molecular Mechanisms of Action. Eur. J. Pharmacol..

[50] Potočnjak I., Marinić J., Batičić L., Šimić L., Broznić D., Domitrović R. (2020). Aucubin Administered by Either Oral or Parenteral Route Protects against Cisplatin-Induced Acute Kidney Injury in Mice. Food Chem. Toxicol..

[51] Forrester S.J., Kikuchi D.S., Hernandes M.S., Xu Q., Griendling K.K. (2018). Reactive Oxygen Species in Metabolic and Inflammatory Signaling. Circ. Res..

[52] Nguepi Tsopmejio I.S., Yuan J., Diao Z., Fan W., Wei J., Zhao C., Li Y., Song H. (2023). Auricularia Polytricha and Flammulina Velutipes Reduce Liver Injury in DSS-Induced Inflammatory Bowel Disease by Improving Inflammation, Oxidative Stress, and Apoptosis through the Regulation of TLR4/NF-ΚB Signaling Pathways. J. Nutr. Biochem..

[53] Zhang W., Liu H.T. (2002). MAPK Signal Pathways in the Regulation of Cell Proliferation in Mammalian Cells. Cell Res..

[54] Ozkok A., Edelstein C.L. (2014). Pathophysiology of Cisplatin-Induced Acute Kidney Injury. BioMed Res. Int..

[55] Mukhopadhyay S., Panda P.K., Sinha N., Das D.N., Bhutia S.K. (2014). Autophagy and Apoptosis: Where Do They Meet?. Apoptosis.

[56] Xu H.-D., Qin Z.-H., Qin Z.-H. (2019). Beclin 1, Bcl-2 and Autophagy. Autophagy: Biology and Diseases.

[57] Potočnjak I., Domitrović R. (2016). Carvacrol Attenuates Acute Kidney Injury Induced by Cisplatin through Suppression of ERK and PI3K/Akt Activation. Food Chem. Toxicol..

[58] Mustonen A.-M., Määttänen M., Kärjä V., Puukka K., Aho J., Saarela S., Nieminen P. (2018). Myo- and Cardiotoxic Effects of the Wild Winter Mushroom (*Flammulina velutipes*) on Mice. Exp. Biol. Med..

[59] Zeng T., Zhang C.-L., Song F.-Y., Zhao X.-L., Yu L.-H., Zhu Z.-P., Xie K.-Q. (2012). PI3K/Akt Pathway Activation Was Involved in Acute Ethanol-Induced Fatty Liver in Mice. Toxicology.

[60] Shackelford C., Long G., Wolf J., Okerberg C., Herbert R. (2002). Qualitative and Quantitative Analysis of Nonneoplastic Lesions in Toxicology Studies. Toxicol. Pathol..

[61] Sun J., Zhang X., Broderick M., Fein H. (2003). Measurement of Nitric Oxide Production in Biological Systems by Using Griess Reaction Assay. Sensors.

[62] Aguilar Diaz De Leon J., Borges C.R. (2020). Evaluation of Oxidative Stress in Biological Samples Using the Thiobarbituric Acid Reactive Substances Assay. J. Vis. Exp..

[63] Rahman I., Kode A., Biswas S.K. (2006). Assay for Quantitative Determination of Glutathione and Glutathione Disulfide Levels Using Enzymatic Recycling Method. Nat. Protoc..

[64] Kurien B., Scofield R. (2006). Western Blotting. Methods.

